# Perceived social support mediates the effect of COVID-19 pandemic on job adaptation disorders of workers: An exploratory cross-sectional study

**DOI:** 10.1097/MD.0000000000037118

**Published:** 2023-02-02

**Authors:** Yongcheng Yao, Jie Tang, Zhenzhen Li, Shuyan Chen, Yuping Li, Hongling Meng, Lingeng Lu

**Affiliations:** a Zhengzhou Normal University, Zhengzhou, Henan, China; b WorldPop, School of Geography and Environmental Science, University of Southampton, Southampton, UK; c Zhengzhou University of Technology, Zhengzhou, Henan, China; d Department of Chronic Disease Epidemiology, Yale School of Public Health, School of Medicine, Yale University, New Haven, Connecticut, USA.

**Keywords:** COVID-19, job adaptation disorders, perceived social support, public health emergencies

## Abstract

COVID-19 lockdown can lead to job adaptation disorders, which are heterogeneous among individuals. The purpose of this study was to explore the association between perceived social support and job adaptation disorders among workers in China during the COVID-19 pandemic. The questionnaires of Psychological Questionnaire for Public Health Emergencies, Multidimensional Scale of Perceived Social Support, Work Attitude Scale were used for this cross-section study via an online survey. The study included 626 employees. Hierarchical regression analysis and Bootstrap method were used to investigate the mediation effect of perceived social support between the emergency and job adaptation disorders. The percentages of the 5 dimensions of depression, neurasthenia, fear, compulsion-anxiety, and hypochondria in workers were 59.7%, 56.1%, 92.3%, 42.0%, and 18.7%, respectively. Social support mediated the relationship between depression, neurasthenia, obsessive-compulsive anxiety and job adaptation disorder, accounting for 18.1%, 16.1%, and 17.5% of the total effect (ab/c), respectively. Perceived social support could alleviate COVID-19 pandemic-related depression, neurasthenia, compulsion-anxiety, and job adaptation disorder in Chinese workers. Improving their perception of social support, workers may better adapt themselves to work in the challenging of the public health emergency during COVID-19 pandemic.

**Strengths and limitations of this study**

► This is the first study with a relatively large sample size to investigate the associations of perceived social support and job adaptation disorders during COVID-19 pandemic in Chinese workers.
► Perceived social support could alleviate the effect of COVID-19 pandemic-related depression, neurasthenia, compulsion-anxiety, and job adaptation disorder in Chinese workers.
► Limitations include that this is a cross-sectional study, and that convenience sampling may have some bias in generalizing to population.

## 1. Introduction

The pandemic of Coronavirus Disease 2019 (COVID-19) has lasted for 2 years since it broke out in early 2020. Many studies show that the outbreak could deteriorate individuals’ mental health.^[1–3]^ The direct threat of highly contagious COVID-19 to physical health, the overflowing media coverage of COVID-19 infection, and the unprecedented large-scale pandemic control measures, such as lockdown, restrictive working and leisure spaces, closure of non-essential businesses and so on all can induce a considerable degree of negative emotions in people.^[4–7]^

In order to effectively prevent and control the pandemic, most residents have been vaccinated. In addition, there are non-drug prevention and control measures such as wearing masks, maintaining social distance, and even suspending work and production. The shutdown of product plants can cause the increase of unemployment. For those still in employment, nucleic acid testing, protective clothing and disinfection have been reinforced as the extra- prevention and control measures. Although strict measurements are taken, fear to contract the disease still looms in individuals. This requires employees to not only overcome the fear of the COVID-19 pandemic, but also quickly adapt to the working environment under the pandemic. Thus, efforts are needed to understand risk factors of job adaptation disorders to provide basis for designing targeted psychological intervention strategies.

Kramer defines reality shock in public emergencies as that employees are prone to a series of negative feelings such as powerlessness, pressure, and frustration due to the unbalance of their own conditions and actual needs in response to environmental changes.^[8]^ On the basis of reality shock theory and combining qualitative research, Duchscher coined the concept of transition shock: when an individual role in work is transformed from a known to an unfamiliar environments, one is affected by the transition of his/her roles, relationships, knowledge and responsibilities, and then experiencing confusion, doubt and unclear orientation.^[9]^ The fear to COVID-19 and unemployment increase the psychological pressure on employees who are still working, putting them under occupational stress, so that they cannot concentrate on their work with feeling fatigue and job adaptation disorders (also known work mal-adjustment).^[10]^

Social support is a potential measurement to intervene job adaptation disorders. A previous study found that perceived social support at the workplace moderates the relationship between different types of conflicts, such as process and task conflicts.^[11]^ Similarly, perceived organizational support has a moderating effect between uncertainty and psychological distress,^[12]^ between role conflict and employees’ satisfaction, and between role ambiguity and leave intention.^[13]^ Social support at work can make employees feel easy, and not alone but they can get support from coworkers and society. Hefner and Eisenberg indicates that perceived social support is more important rather than actual social support in an individual mental health.^[14]^ However, the role of perceived social support pertaining to work attitude during the COVID-19 pandemic and its mediating effect need further validation in Chinese workers.

The mediating effect of social support represents a mechanism of action to explain how the COVID-19 pandemic affects job adaptation disorders, thereby providing theoretical support for practical interventions.^[15–18]^ This study aims to explore the mediating effect of worker social support between public health emergencies and job adaptation disorders. We hypothesized that employees had different psychological responses when they faced the COVID-19 pandemic in China, and that job adaptation disorders were associated with social support.

## 2. Participants and methods

### 2.1. Participants

#### 2.1.1. Sample size and the sampling technique.

We estimated the sample size (n) for this study based on a single population proportion formula, N = [u_α_^2^π(1-π)]/δ^2^ with the assumption of δ = 0.03, α = 0.05, u_α_ = 1.96 and the overall comprehensive estimate of the incidence of stress symptoms in workers during the COVID-19 epidemic is 17.52% (*π *= 0.175).^[19]^ A total number of 617 participants was needed.

#### 2.1.2. The convenience sampling method was applied in this study.

During the period from October to December 2021, a cross-section survey was conducted on workers using online questionnaires via the platform of “Questionnaire Star” in Zhengzhou, the capital of Henan Province, China. The inclusion criteria were the participants with working length > 1 year, and no diagnosed physical and mental illnesses. The informed consent was obtained from all participants. The protocol for this study was reviewed and approved by the Ethics Committee of Zhengzhou Normal University (ref. no. ZZNU-2021-006). The enrollment of the participants complied with the 1964 Helsinki declaration and its later amendments or comparable ethical standards.

A total of 626 valid questionnaires were collected via an online system of the questionnaire star survey platform. Of them, 183 (29.2%) were men and 443 (70.8%) were women. Participants were 19 to 60 years old, with an average age of 38.4 years. The basic demographic information include age, sex, marital status, length of service (working length), stay up late, and exercise frequent (at least half an hour each time).

### 2.2. Method

#### 2.2.1. Psychological response.

The Psychological Questionnaires for Emergent Events of Public Health (PQEEPH) questionnaire was used to measure the psychological response of the participants to public health emergencies.^[20]^ This is a widely used questionnaire for individuals’ psychological status evaluation during public health emergencies.^[21,22]^ There are 25 items in the PQEEPH with 5 dimensions: depression, neurasthenia, fear, compulsion-anxiety, and hypochondria. A 4-point scale from 0 to 3 was assigned for the extent (none, mild, moderate, or severe) and frequency (occasionally, sometimes, regularly, or always) of the emotional reaction. The score for each dimension is the ratio of the total score of the dimension and the number of items. A higher score indicates a severe psychological symptom. According to the depression dimension score, depression with a score >0 is a positive result, and it was classified into mild (≤1), moderate (1~2), and severe (>2).^[22]^ The Cronbach alpha of the 5 dimensions (depression, fear, neurasthenia, compulsion-anxiety, and hypochondria) was 0.871, 0.750, 0.663, 0.655, and 0.425, respectively.

#### 2.2.2. Perceived Social Support (PSS).

The Multidimensional Scale of Perceived Social Support was used to measure the social support that the participants could obtain, which was developed by Zimet^[23]^ in 1988. The MSPSS is composed of 12 items, each of which rates on a 7-point Likert-type scale, where 1 indicates “very strongly disagree” and 7 indicates “very strongly agree.” The range of summed score of the MSPSS is between 12 and 84. A positive association exists between the score and both better social support and satisfaction. 3 dimensions are in the scale: family support (4 items), friend support (4 items) and other support (e.g., relatives and colleagues). The scale reflects the individual perceived social support. The MSPSS in Chinese version has been widely used in China to assess an individual perceived social support with a good measure.^[24]^ The Cronbach alpha in this study was 0.954.

#### 2.2.3. Job adaptation disorders (JAD).

The Work Attitude Scale was used to measure the employee motivation and attitudes toward work.^[25]^ There are a total of 37 items each with a score of either 0 or 1. The sum of item scores is the original score, which is converted into T score (T = 50 + 10 × (the original score of the subject - the average score of the group)/ the standard deviation of the score in the group. High scores on the scale indicate a lack of work motivation and maladjustment. The Cronbach α coefficient of the scale in this study was 0.861.

### 2.3. Statistical analysis

All analyses were performed with the software SPSS 18.0. The mediation analyses were conducted using the PROCESS plugin for SPSS.^[26]^ The numerical variables in an approximate normal distribution with normality test (the absolute values of Skew and kurtosis are both <1) were expressed in mean ± SD to represent its average and standard deviation. All numerical variables were normal distributed except 5 dimensions of PQEEPH, which described as the median (*M*) and quartiles (*P*_*25*_, *P*_*75*_). Either t-test or Analysis of Variance (ANOVA) was used to analyze the differences between the groups, the Bonferroni method was used for the correction of multiple comparisons, and the Dunnett^,^ sT3 method was used when the variance was uneven. Non-parametric Mann–Whitney *U* Test and Kruskal–Wallis H Test were used to compare the 5 dimensions of PQEEPH with different demographic variables. The relationship between PQEEPH, PSS and JAD were analyzed using Spearman correlation, and described by Spearman correlation coefficient (*r*_*s*_); the relationship between PSS and JAD was analyzed using Pearson correlation and described by Pearson correlation coefficient (*r*). A *P* value <.05 is considered statistically significant.

Hierarchical regression and Bootstrap methods were used to examine the mediating effect of PSS between PQEEPH and JAD. The strength of the indirect effects was assessed using the PROCESS plugin for SPSS,^[26]^ in which total, direct and indirect effects are analyzed for significance.^[27–29]^ A point estimate and 95% confidence intervals (CI) of the indirect effect ab (a × b) was calculated via bootstrap approach with 5000 bootstrapped samples, as suggested by.^[28]^

## 3. Results

### 3.1. Common-method variance test

The Harman single factor test was used to measure the common-method variance. There was no severe common-method variance in this study. The eigenvalues of 28 factors were >1, and the first factor explained 18.13% of the variance, which is less than the criterion of 40%.^[30]^

### 3.2. The score of the psychological questionnaire on public health emergencies

The detection rates of the depression, neurasthenia, fear, compulsion-anxiety, and hypochondria in workers during the pandemic COVID-19 were 59.7% (374), 51.6% (323), 92.3% (578), and 42.0% (263), and 18.7% (117) respectively.

### 3.3. Associations of public health emergencies, social support, and job adaptation disorders of workers

Table 1 shows the associations of demographic factors with public health emergencies, social support, and job adaptation disorders in Chinese workers in Henan. The neurasthenia and fear scores in male workers were lower than women (*P < *.01). The neurasthenia score in married workers was higher than single (*P < *.05). There was a significant association between depression, neurasthenia, and compulsive-anxiety among age (*P < *.05). Post hoc test results showed statistically significant differences in the depression of workers among the 3 age groups (*P < *.05). The depression score in the workers aging < 35 years was the lowest, and aging 41~ years was the highest. The workers aging < 35 years scored significantly higher on the neurasthenia and compulsive-anxiety subscale than those aging 41~ years (*P < *.01). There were statistically significant differences in the depression and neurasthenia for workers with the different working lengths in years (*P < *.01). Post hoc test results showed that the workers with < 11 years and 11~ years of service scored significantly lower on the depression subscale than those with 21~ years of service (*P < *.001). The workers with < 11 years of service scored significantly lower on the neurasthenia subscale than those with 21~ years of service (*P < *.01).

**Table 1:** Associations of demographic variables with Psychological response [M(*P*_25_, *P*_75_)], PSS and JAD(mean ± SD).

Variable	n	Depression	Neurasthenia	Fear	Com-An	Hyp	PSS	JAD
Sex								
Male	183	0.17 (0.00,0.67)	0.00 (0.00,0.40)	0.50 (0.33,1.00)	0.00 (0.00,0.17)	0.00 (0.00,0.00)	66.27 ± 13.74	51.02 ± 11.25
Female	443	0.17 (0.00,0.67)	0.20 (0.00,0.60)	0.67 (0.50,1.17)	0.00 (0.00,0.17)	0.00 (0.00,0.00)	65.42 ± 13.23	49.57 ± 9.42
* Z/t*		–0.186	–3.278**	–3.119**	–0.130	–0.333	0.723	1.538
Marital status								
Single	69	0.00 (0.00,0.67)	0.00 (0.00,0.20)	0.67 (0.17,1.00)	0.00 (0.00,0.17)	0.00 (0.00,0.00)	66.35 ± 13.15	50.08 ± 10.86
Married	557	0.17 (0.00,0.67)	0.20 (0.00,0.40)	0.67 (0.33,1.00)	0.00 (0.00,0.17)	0.00 (0.00,0.00)	65.58 ± 13.42	49.98 ± 9.90
* Z/t*		–1.627	–2.344^*^	–1.221	–0.339	–0.570	0.448	0.075
Age								
<35	182	0.00 (0.00,0.50)	0.00 (0.00,0.40)	0.67 (0.17,1.00)	0.00 (0.00,0.17)	0.00 (0.00,0.00)	65.48 ± 13.91	50.01 ± 10.73
35~	213	0.17 (0.00,0.67)	0.20 (0.00,0.40)	0.67 (0.50,1.00)	0.00 (0.00,0.17)	0.00 (0.00,0.00)	64.41 ± 13.74	50.34 ± 10.20
41~	231	0.33 (0.00,0.83)	0.20 (0.00,0.60)	0.67 (0.33,1.00)	0.00 (0.00,0.33)	0.00 (0.00,0.00)	66.97 ± 12.52	49.66 ± 9.22
* H/F*		26.702***	10.525**	5.124	7.272*	1.074	2.062	0.249
Working length (yr)								
<11	204	0.17 (0.00,0.63)	0.00 (0.00,0.40)	0.67 (0.33,1.00)	0.00 (0.00,0.17)	0.00 (0.00,0.00)	64.63 ± 13.61	50.07 ± 10.51
11~	242	0.17 (0.00,0.67)	0.20 (0.00,0.40)	0.67 (0.46,1.00)	0.00 (0.00,0.17)	0.00 (0.00,0.00)	65.79 ± 13.83	49.85 ± 10.08
21~	180	0.50 (0.00,0.83)	0.20 (0.00,0.60)	0.67 (0.38,1.13)	0.00 (0.00,0.33)	0.00 (0.00,0.00)	66.67 ± 12.46	50.10 ± 9.33
* H/F*		25.869***	9.656**	1.960	5.395	1.082	1.124	0.043
Exercise								
Frequently	233	0.17 (0.00,0.67)	0.00 (0.00,0.40)	0.67 (0.33,1.00)	0.00 (0.00,0.17)	0.00 (0.00,0.00)	67.94 ± 12.19	49.48 ± 10.59
Sometimes	283	0.17 (0.00,0.67)	0.00 (0.00,0.40)	0.67 (0.33,1.00)	0.00 (0.00,0.17)	0.00 (0.00,0.00)	65.42 ± 12.50	49.53 ± 9.44
Frequently	110	0.42 (0.00,0.83)	0.20 (0.00,0.80)	0.67 (0.33,1.17)	0.00 (0.00,0.33)	0.00 (0.00,0.13)	61.47 ± 16.62	52.28 ± 9.89
* H/F*		9.856**	20.142***	1.286	5.218	3.340	9.057***	3.523*
Stay up								
Never	115	0.00 (0.00,0.33)	0.00 (0.00,0.40)	0.50 (0.33,0.83)	0.00 (0.00,0.17)	0.00 (0.00,0.00)	67.74 ± 13.66	47.96 ± 8.95
Seldom	166	0.17 (0.00,0.67)	0.00 (0.00,0.40)	0.58 (0.33,1.00)	0.00 (0.00,0.17)	0.00 (0.00,0.00)	66.87 ± 12.09	49.17 ± 9.89
Sometimes	223	0.17 (0.00,0.67)	0.20 (0.00,0.60)	0.83 (0.50,1.00)	0.00 (0.00,0.17)	0.00 (0.00,0.00)	65.17 ± 13.05	49.97 ± 9.97
Frequently	122	0.67 (0.17,1.00)	0.20 (0.00,0.80)	0.67 (0.50,1.17)	0.08 (0.00,0.33)	0.00 (0.00,0.00)	62.98 ± 14.95	53.07 ± 10.52
* H/F*		50.149***	24.330***	17.077**	6.463	4.046	3.142*	5.958**

Com-An = compulsion-anxiety, Hyp = hypochondria, PSS = Perceived Social Support, JAD = job adaptation disorders.

* *P* < .05

** *P* < .01

*** *P* < .001.

Exercise frequency significantly associated with depression, neurasthenia, social support, and job adaptation disorders (*P < *.05). Further pairwise comparisons showed that the depression score of the workers with regular exercise was lower than the non-exercise group (*P < *.01), the neurasthenia score of the workers with regular or occasional exercise was lower than the non-exercise group (*P < *.001), and the social support scores of the workers with regular exercise were higher than the non-exercise group (*P < *.01), and the job adaptation disorder scores of the workers who exercised regularly or occasionally were lower than the non-exercise group (*P < *.05).

Stay up significantly associated with depression, neurasthenia, fear, social support, and job adaptation disorders (*P < *.01). Post hoc test results showed that the scores of depression for the workers with no stay up was the lowest (*P < *.05), while the scores of depression for those with frequent stay up was the highest (*P < *.001). The scores of neurasthenia and fear of workers who often (frequent or sometimes) stay up late are higher than those who rarely (never or seldom) stay up late (*P < *.05). The social support scores of workers who stayed up late were lower than those who rarely stayed up late (*P < *.05). The workers who often stay up late have the highest score of job adaptation disorder (*P < *.01).

### 3.4. Correlation between emergent events of public health, perceived social support and job adaptation disorders

Table 2 shows the correlation between emergent events of public health, social support, and job adaptation disorders. Pearson correlation results demonstrated that a significantly negative correlation between social support and depression, neurasthenia, fear, compulsive-anxiety (*r = *−0.225, −0.323, −0.096, −0.167, *P < *.01). A significantly positive correlation was also found between job adaptation disorders and all dimensions of emergent events of public health (*R = *0.361, 0.441, 0.137, 0.335, and 0.146, respectively, *P < *.01). A significantly negative correlation between social support and job adaptation disorders (*r = *−0.357, *P < *.01).

**Table 2:** Pearson correlation between psychological response, PSS and JAD(n = 626)

Variable	Depression	Neurasthenia	Fear	Com-An	Hyp	PSS	JAD
Depression	1.000						
Neurasthenia	0.536**	1.000					
Fear	0.327**	0.288**	1.000				
Com-An	0.510**	0.541**	0.329**	1.000			
Hyp	0.104**	0.340**	0.316**	0.325**	1.000		
PSS	–0.225**	–0.323**	–0.096*	–0.167**	–0.053	1.000	
JAD	0.361**	0.441**	0.137**	0.335**	0.146**	–0.357^**^	1.000

Com-An = compulsion-anxiety, Hyp = hypochondria, PSS = Perceived Social Support, JAD = job adaptation disorders.

* *P*<.05

** *P*<.01.

### 3.5. Mediating effect of social support between emergent events of public health and job adaptation disorders

Taking job adaptation disorders score as the dependent variable, sex, age, and working length as covariates, Emergent Events of Public Health and social support as independent variables were stepwise incorporated into the regression equation, and the results are illustrated in Table 3. In the model 1, when only covariate variables were included, there was not statistically significant of the model. Under controlling the influence of sex, age, and working length, depression and neurasthenia had a positive impact on job adaptation disorders after the inclusion of the 5 dimensions of Emergent Events of Public Health (β = 2.785 and 8.820, respectively, *P < *.05), which explained 23.2% of the variance of job adaptation disorders.

**Table 3:** Hierarchical regression results of risk factors of Job adaptation disorders in Chinese workers in Henan during the pandemic COVID-19.

Variable	Model 1		Model 2		Model 3	
	β	*P*	*β*	*P*	*β*	*P*
Covariates						
Sex	-1.453	0.100	-2.382	0.003	-2.321	.003
Age	-0.038	0.688	-0.143	0.087	-0.108	.185
Working length (yr)	0.006	0.938	0.015	0.830	0.010	.885
Depression			2.785	0.007	2.535	.012
Neurasthenia			8.820	<0.001	7.010	<.001
Fear			-1.131	0.159	-0.981	.209
Com-An			1.673	0.383	1.771	.341
Hyp			0.320	0.812	0.778	.552
PSS					-0.169	<.001
* F*	1.022	0.382	23.327	<0.001	26.306	<.001
* R* ^2^	0.005		0.232		0.278	
△*R*^2^	0.005		0.227		0.045	

Com-An = compulsion-anxiety, Hyp = hypochondria, PSS = Perceived Sociel Support.

However, in the model 3 of the introduction of social support, the explained variance increased by 4.5%, which had a negative impact on job adaptation disorders (β* = *−0.169, *P < *.001), while the absolute value of the standardized regression coefficient of depression decreased from 2.875 to 2.535, the absolute value of the standardized regression coefficient of neurasthenia decreased from 8.820 to 7.010. The standardized regression coefficients of depression and neurasthenia were all statistically significant, suggesting that social support might play a partial mediating role in the relationship between depression, neurasthenia, and job adaptation disorder.

The Bootstrap method was used to analyze the mediating effect of the 5 dimensions of public health emergencies. Social support played a partial mediating role in the relationship between depression, neurasthenia, obsessive anxiety and job adaptation disorder, and the mediating effect accounted for 18.1%, 16.1%, and 17.5% of the total effect (ab/c), respectively. In contrast, social support had no mediating effect on the relationship between fear, hypochondria, and job adaptation disorder (Table 4).

**Table 4:** Analysis of the mediating effect of Perceived social support between psychological response and job adaptation disorders in workers during the pandemic COVID-19 (Bootstrap).

Variable	a	b	c	c	ab (95%CI)
Depression	–6.626***	–0.213***	7.780***	6.368***	1.412(0.842, 2.077)
Neurasthenia	–10.484***	–0.172***	11.194***	9.388***	1.805 (1.105, 2.639)
Fear	–1.825	–0.261***	2.657***	2.181**	0.476 (-0.093, 1.075)
Com-An	–9.967***	–0.224***	12.756***	10.527***	2.229 (1.193, 3.393)
Hyp	–1.695	–0.263***	4.756***	4.311***	0.446 (-0.469, 1.434)

Com-An = compulsion-anxiety, Hyp = hypochondria, a = the effect of the independent variable on Perceived social support, b = the effect of perceived social support on Job adaptation disorders, c = he total effect of the independent variable on job adaptation disorders, c’ = the direct effect of the independent variable on Job adaptation disorders after the introduction of perceived social support, a × b = the mediating effect of perceived social support between psychological stress and job adaptation disorders.

*P<.05

** *P*<.01

*** *P*<.001.

## 4. Discussion

In this study, we found that the psychological stress response of Chinese workers in Henan Province was mainly mild during the pandemic COVID-19, and the positive rate of each dimension was lower than the previous report as by Jinman.^[31]^ The results of this study show that women had more severe psychological reactions, which may be due to women being relatively vulnerable at work and worrying about unemployment. Depression, neurasthenia, obsessive anxiety and fear among the workers in the 41-age group, and depression in the 21- age group are more pronounced compared to other groups, probably because these groups often shoulder the double burden of family and occupation, and once their health is threatened, If the life and work chain cannot be balanced, it is more likely to generate negative emotions.^[31]^ Workers with regular exercise usually have a wide range of contacts and understand social support better. Via exercise, an individual not only maintains physical health, but also accumulates many social relationships through communication with people. Perceived social support is inversely related to work adjustment disorder. Workers who lack exercise generally had a poor work adaptability. In addition, the depressive and neurasthenic responses of workers who exercise regularly are lower, while the depression, neurasthenia, and fear responses for those who often stay up late are more pronounced. Exercise but not stay up can maintain good physical fitness and mental health. In contrast, stay up will increase psychological burdens such as depression. Workers who often stay up late may show a certain degree of discomfort in working hours due to unreasonable time allocation in their daily life.

In this study, we found that the dimensions of public health emergencies were positively correlated with job adaptation disorders. In the hierarchical regression analysis, when the 5 dimensions of public health emergencies were included at the same time, the regression coefficients, and models of the dimensions of depression and neurasthenia were statistically significant, and the explained variance increased by 22.2%, indicating that depression and neurosis frailty is a stronger predictor of work adaptability impairment. The global spread of the COVID-19 has not only brought about a global public health crisis, but also brought far-reaching impact and losses to the world economy.^[32]^ The direct impact of the domestic epidemic on the economy is to cause the suspension of flights and operations, and the suspension of production and production of enterprises. As a result, employees are unemployed or working intermittently, which severely affects the economic income of families. Even workers always maintain a sense of crisis and worry about unknown reasons. The recurrence of the epidemic has affected work. In such an environment, workers will inevitably experience dependence and discomfort on work, difficulty in concentrating, mental stress, and often feel pressured, which will cause workers to adapt themselves in difficulties to work obstacles.

Our study also found that perceived social support was negatively correlated with depression, neurasthenia, fear, and obsessive-compulsive anxiety dimensions of public health emergencies, and negatively correlated with work adjustment disorder. When the 5 dimensions of public health emergencies were separately included, the results of the Bootstrap method suggested that the mediating effect of perceived social support in the relationship between depression, neurasthenia, fear, obsessive-compulsive anxiety, and work adaptability disorder was statistically significant. Understanding social support is the subjectively experienced social support, which refers to the emotional experience and satisfaction that an individual feels respected, supported, and understood in society. Pushkarev makes the statement that perceived social support is an individual expectation and evaluation of social support, and is a belief in possible social support.^[33]^ Comprehension of social support is a positive factor of job adaptation, indicating that job adaptation is enhanced with the improvement of the level of perceived social support. The higher the perceived social support, the better the job adaptation. When workers encounter emergencies such as stress or danger at work, if they have good social support and can effectively use these supports, they will be able to better relieve psychological pressure and actively respond to and deal with these events and scenarios.^[34]^ Therefore, by increasing perceived social support, job adaptation disorders caused by public health emergencies can be potentially alleviated.

Limitations exist in this study. First, this survey was a cross-section study, the investigated factors may be accompanied rather than a causal relationship. Thus, causal inference cannot be made from this study. Second, in this study, convenience sampling approach was applied to collect data, which is more convenient with a low cost, and a high response rate in comparison to random sampling. However, such sampling approach may have bias in representative, cautions should be paid in generalizing and interpreting the results.

In summary, this study found that perceived social support mediated the effect of COVID-19 on job adaptation disorders in Chinese workers. The more social support workers perceive, the better their mental health is, which in turn motivates workers positive and enthusiasm to work. The more social support workers perceive, the more helpful they are to perceive their own worth and abilities, and to understand the significance of work. This study shows that in the face of the pandemic COVID-19, the mental health of Chinese workers in Henan has been greatly affected. Depression and neurasthenia significantly increased job adaptation disorders in Chinese workers during COVID-19 pandemic. The findings indicate that social support may improve mental health, alleviating job adaptation disorders in Chinese workers during COVID-19 pandemic. Thus, policymakers may make different strategies to let workers feel warm and comfortable, and know they are not alone.

## Acknowledgments

The authors thank all participants in this study.

## Author contributions

**Conceptualization:** Yongcheng Yao.

**Data curation:** Jie Tang, Shuyan Chen.

**Formal analysis:** Yuping Li.

**Funding acquisition:** Yongcheng Yao.

**Investigation:** Zhenzhen Li, Hongling Meng.

**Methodology:** Zhenzhen Li, Lingeng Lu.

**Project administration:** Zhenzhen Li, Yuping Li.

**Resources:** Jie Tang.

**Software:** Jie Tang.

**Supervision:** Yuping Li, Hongling Meng, Lingeng Lu.

**Validation:** Shuyan Chen.

**Visualization:** Shuyan Chen, Yuping Li, Hongling Meng.

**Writing – original draft:** Yongcheng Yao.

**Writing – review & editing:** Yongcheng Yao, Lingeng Lu.
